# A Review on Techniques and Biomaterials Used in 3D Bioprinting

**DOI:** 10.7759/cureus.28463

**Published:** 2022-08-27

**Authors:** Ankita Sachdev, Sourya Acharya, Tejas Gadodia, Samarth Shukla, Harshita J, Chinmay Akre, Mansi Khare, Shreyash Huse

**Affiliations:** 1 Department of Surgery, Datta Meghe Institute of Medical Sciences (Deemed to be University), Wardha, IND; 2 Department of Medicine, Datta Meghe Institute of Medical Sciences (Deemed to be University), Wardha, IND; 3 Department of Pathology, Datta Meghe Institute of Medical Sciences (Deemed to be University), Wardha, IND

**Keywords:** fixed deposition modelling, three-dimensional bioprinting, synthetic polymers, selective laser sintering, inkjet, bioink, polymers, conduit, printing, organ

## Abstract

Three-dimensional (3D) bioprinting is a cutting-edge technology that has come to light recently and shows a promising potential whose progress will change the face of medicine. This article reviews the most commonly used techniques and biomaterials for 3D bioprinting. We will also look at the advantages and limitations of various techniques and biomaterials and get a comparative idea about them. In addition, we will also look at the recent applications of these techniques in different industries. This article aims to get a basic idea of the techniques and biomaterials used in 3D bioprinting, their advantages and limitations, and their recent applications in various fields.

## Introduction and background

Three-dimensional (3D) printing of biological material is a revolutionary technology through which we can print various materials ranging from simple muscle tissue, neural tissue, and cartilage, to an entire organ. In this process, we first construct a 3D model of the structure we want to print using patient's scans such as X-ray, CT, or MRI, which will then be printed in a layer-by-layer model taking care of every microscopic as well as macroscopic detail of the tissue. This model is then printed in a layer-by-layer fashion, which is then further processed to hold it together to function as a single unit [1]. While printing a particular structure, we need to keep in mind the properties of biomaterials used, such as biocompatibility, strength, stability, and immunogenicity, before selecting the correct biomaterial [2].

Bioprinting is not a single-step process; it involves various complex processes to print customized 3D structures for the patient, such as designing the structure with the help of computers using the patient's radiological imaging reports and then prototyping using a technique known as solid free form fabrication, which will take care of every microscopic as well as macroscopic detail of the tissue. With the progress in bioprinting technology and the qualities of biomaterials, 3D bioprinting can lead to various advantages in the short and long run. Although, at present, it seems scary to a normal person to think about having a printed organ in his own body, if this technology succeeds, it can save so many people waiting for years for organs [3,4].

Other uses of 3D bioprinting are the treatment of burn wounds using artificial skin, bioprinting of bones and cartilage, drug testing, preparing diseased tissue models to check the treatment's efficacy before actually giving it to a patient, bladder implants, and heart valve implants. Besides so many advantages that we can have from 3D bioprinting, there are many challenges ahead of us, such as the technology being too expensive. This technology will only be advantageous for only a few people, leaving behind the poor who will have to wait for a donor. Also, as this technology is not yet so advanced, that makes it a very risky procedure as we still do not have all the information about the types of complications that can occur from this procedure. Also, there is still a long road ahead of us, which requires years of research to make this procedure successful [5].

The main goal of 3D bioprinting is to replace the non-functioning or defective tissue/organ with the new bioprinted one, which will function the same as the native organ structurally and functionally. This bioprinted tissue must know how to regenerate and differentiate on its own when implanted inside the patient's body. With the proper use of technology and the correct type of biomaterial, adequate tissue can be printed, which will perform all these required functions; therefore, adequate research in the field of biomaterials is needed to find the correct material that can work as native tissue. In this review article, commonly used bioprinting technology, their application, advantages, and limitations, along with types of biomaterials used in the field of 3D printing (both natural and synthetic) and their advantages and limitations have been discussed, as well as their application in the various industries [6].

## Review

Typically used techniques in bioprinting 

**Figure 1:**
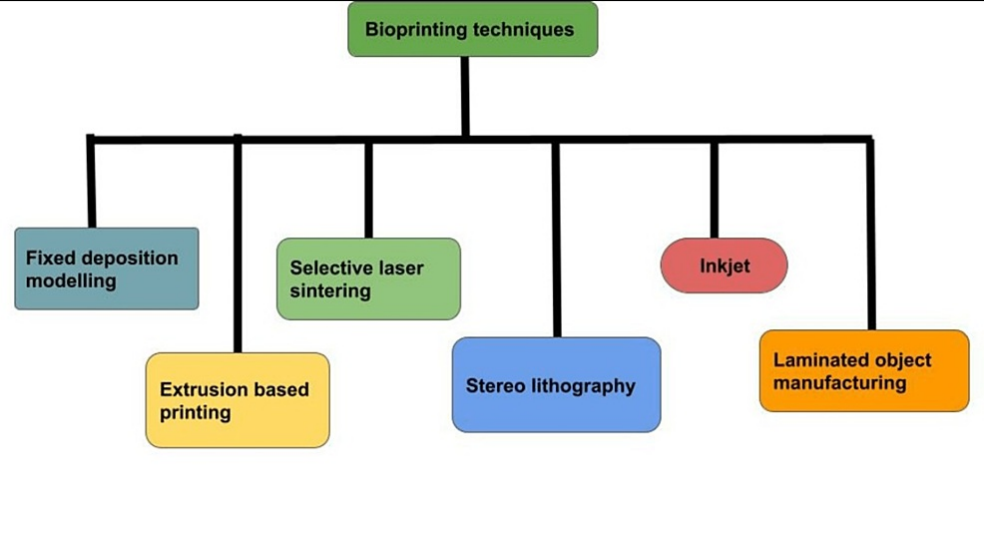
Different Types of Techniques Used in Bioprinting

Among all the types of techniques used in bioprinting, the most commonly used methods are described in Figure 1 and the biomaterials used in them are described in Table 1.

**Table 1:** Summary of Commonly Used Techniques in Bioprinting PEG, polyethylene glycol; PVA, polyvinyl alcohol; TCP, tricalcium phosphate; UV; ultraviolet; 3D, three-dimensional

Techniques	Procedures	Biomaterials	Applications
Fixed deposition modelling	Heat-sensitive plastic filaments are melted down and arranged in a layer-by-layer fashion to build a 3D object [2]	Nylon, PVA, polycarbonate	Regeneration of cartilage tissues, bone tissue; delivery of antibiotics; prosthetics [3]
Extrusion-based printing	Extrusion of the material using pressure through the nozzle of the printer is done to form the desired shape [2]	Collagen, hyaluronic acid, alginate, PEG, gelatin, chitosan	Aortic valve; neural tissue; muscle tissue; bones; implants [2]
Selective laser sintering	Solid 3D structures are formed using a powder arranged in a layer-by-layer fashion using a high-power laser [4]	Ceramics, metals, polyamide	Drug delivery; tissue engineering
Stereolithography	Photopolymers of high sensitivity are bound together using a beam of UV laser, heat, or electron beam	Photopolymers	Medical models and prototypes
Inkjet	Alternate powder and liquid binding material layers are added in a layer-by-layer fashion [5]	Hydroxyapatite, Alpha- TCP, beta -TCP, PVA, PEG, PEG hydrogel	Printing of biomolecules such as protein and nucleic acid
Laminated object manufacturing	Thin sheets are coated with adherent material, glued together in a layer-by-layer fashion, and then cut into the desired shape using a laser or metal cutter [2]	Metals, Plastic, Paper	Prototypes

The advantages and limitations of the methods are illustrated in Table 2.

**Table 2:** Summary of Advantages and Limitations of Different Bioprinting Techniques

Techniques	Advantages	Limitations
Fixed deposition modelling	Low cost, quick processing, easy to operate, high porous materials can be made	Less compatibility, high temperature destroys the material, lack of mechanical strength, only thermoplastics can be used
Extrusion-based printing	Long viability, can print highly dense material, low cost	Pressure may affect cell viability, cannot print complex tissue
Selective laser sintering	Good support offered from a powder bed, many types of materials can be used	Highly expensive, printers are large and complex to install, process is slow
Stereolithography	High resolution, high viscous material can be printed [3].	Ultraviolet rays used are toxic and make skin cancer-prone, slow process, cell viability is short
Inkjet	Quick processing, high resolution, long viability, more compatible, multicolor printing is possible [5]	Low mechanical strength, nozzle gets blocked frequently because of the highly dense material used
Laminated object manufacturing	Low cost, quick processing, easy to operate	Difficulty in manufacturing complex tissues

Biomaterials

**Figure 2:**
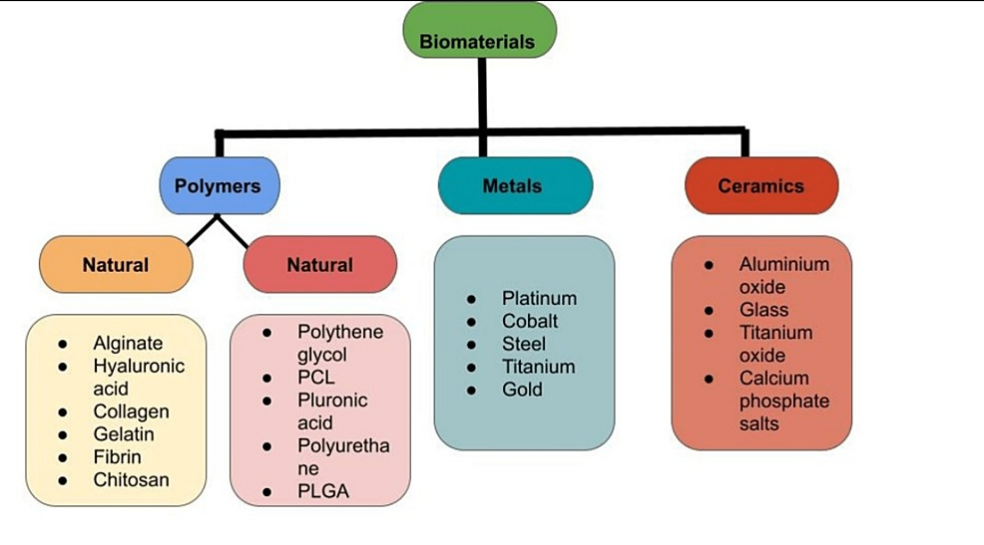
Classification of the Biomaterials Used in Bioprinting PCL, polycaprolactone; PLGA, polylactic-co-galactic acid

Typically used biomaterials used in 3D printing have been illustrated in Figure 2.

Natural Polymers

Naturally occurring polymers can be derived using various physical, biochemical, or chemical methods. Natural polymers are compatible, can hold the fluid, and can be easily dissolved in different solvents such as phosphate buffers and cell culture solutions, making them more tissue friendly. Due to these qualities, it is possible to print it in a layer-by-layer manner, producing a model that will mimic a natural organ if placed in a stable environment [6,7]. One of the critical properties of these naturally occurring polymers is that when provided with a controlled environment such as normal temperature, adequate water, and proper medium to grow, they can mimic cells or tissue, undergo proliferation, maturation, and differentiation, and coordinate with surrounding structures [8-10]. One major drawback of natural polymer is that all these activities are majorly affected if the surrounding environment becomes unstable, such as an increase in temperature, dehydration, or the nature of the solvent in which it is dissolved. Some commonly used natural polymers are alginate, gelatin, collagen, chitosan, and hyaluronic acid, and are described below.

Alginate

**Figure 3:**
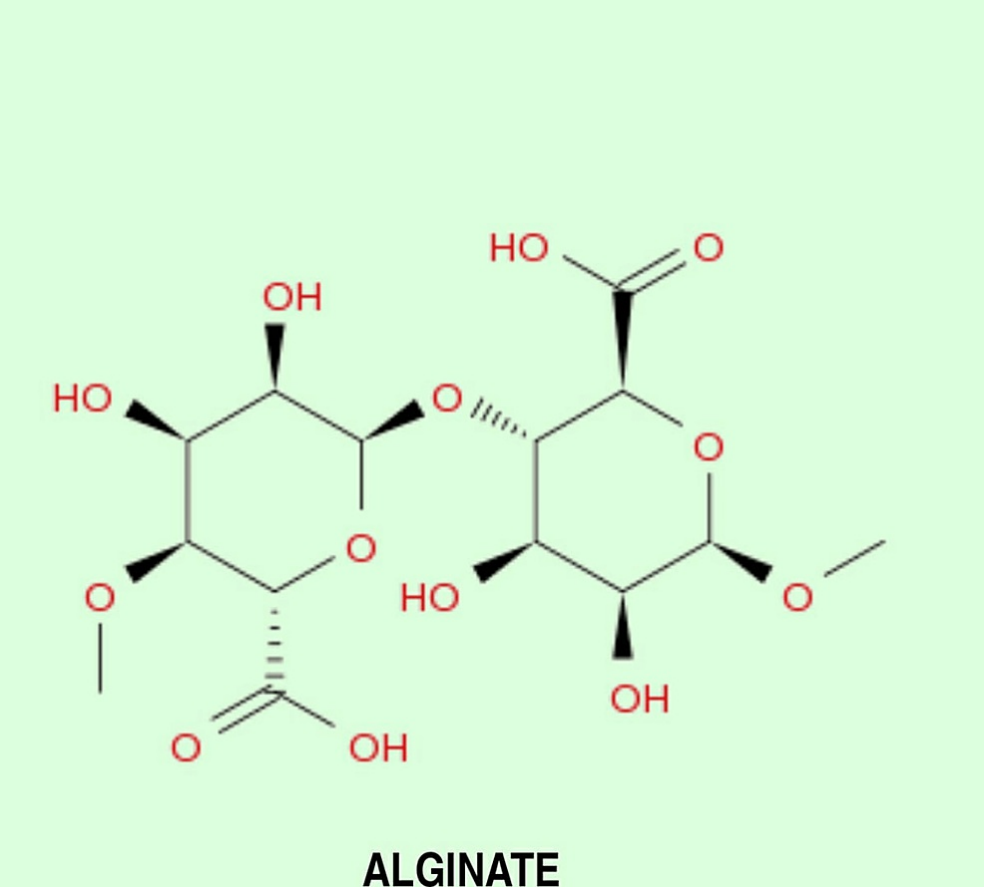
Chemical Structure of Alginate

Alginate is derived from the cell walls of Phaeophyceae (brown algae) and is used in the form of salts of alginic acid. Wang first used alginate in the form of sodium alginate, but the problem faced was its gelation point, which is 0°C, while 3D bioprinting was done at room temperature; therefore, it was crosslinked with other metals such as calcium and barium to increase its compatibility and mechanical strength [11]. An important thing to take care of while using alginate is to use it in adequate concentration because if used in less concentration, the model's strength is majorly affected. All the activities such as proliferation, growth, and maturation are affected if used in high concentration. Therefore, it is crucial to use the proper guidelines regarding the concentration of alginate to be used for 3D bioprinting. Also, as alginate shows the property of delayed degradation, it is recommended to use alginate in an oxidized form, which is expected to show increased degradability and will be more suited for 3D bioprinting [12]. The chemical structure of alginate is given in Figure 3.

Hyaluronic Acid

**Figure 4:**
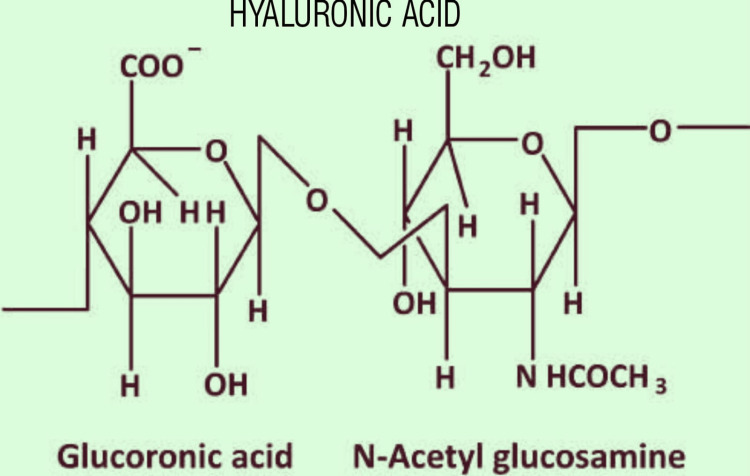
Chemical Structure of Hyaluronic Acid

Hyaluronic acid is an integral part of the extracellular matrix, which plays a major role in the proliferation of cells and angiogenesis. Due to its high cell adhesive property and water-absorbing quality, it can be used to change the viscosity of other polymers such as gelatin. As with other natural polymers, hyaluronic acid is crosslinked with synthetic polymers to increase its compatibility. One example is the crosslinking of hyaluronic acid with methyl acrylate forming a rigid non-biodegradable polymer known as HAMA (hyaluronic acid methylacrylate) [13,14]. Another polymer formed via crosslinking is GeIMA which, when used in combination with HAMA (HAMA-GeIMA), will increase its mechanical strength and compatibility. It has been proven that the 1:4 ratio of GeIMA:HAMA is an adequate ratio to improve the compatibility of the polymer formed (np 101). As this Combination shows superior qualities, it has been applied in the bioprinting of musculoskeletal, cardiac, and neural tissues [15]. The chemical structure of Hyaluronic acid is given in Figure 4.

Collagen

**Figure 5:**
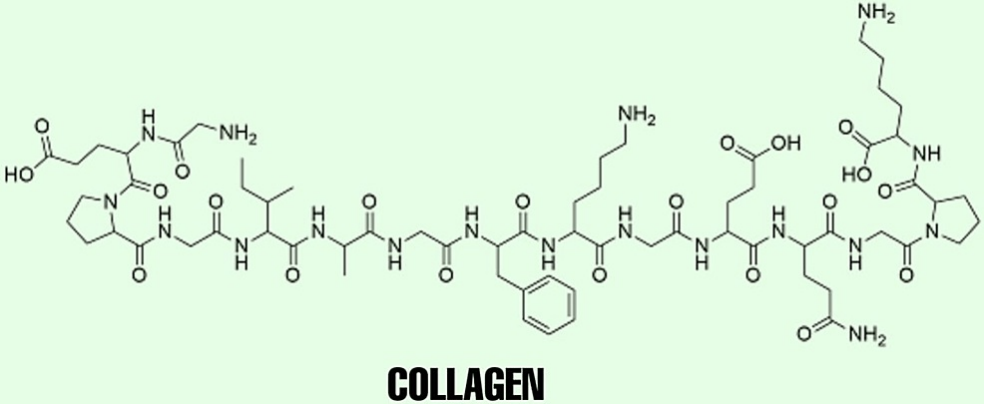
Chemical Structure of Collagen

Collagen is widely known to support the skin, ligaments, bone, tendon, and cartilage due to its resistance and toughness. Type 1 and type 2 are most often used in musculoskeletal repair using 3D printed models. Collagen has been observed to promote proliferation, maturation, and differentiation of bone and cartilage cells [16]. As seen in other polymers, using collagen in bioprinting is best done when combined with other polymers to increase its viscosity and decrease its degradation compared to using collagen only. It is commonly crosslinked with alginate, agarose, hyaluronic acid, and fibrin [17]. To increase its compatibility, collagen has also been crosslinked with heparin sulfate and polyurethane for printing conduits, which can help nerve repair [18-19]. However, the drawback of using collagen is its easy solubility in acids, making it temperature- and pH-dependent. Also, the rapid degradation of collagen by collagenase and metalloproteinase in the body makes it difficult to use [20]. The chemical structure of collagen is given in Figure 5.

Gelatin

**Figure 6:**
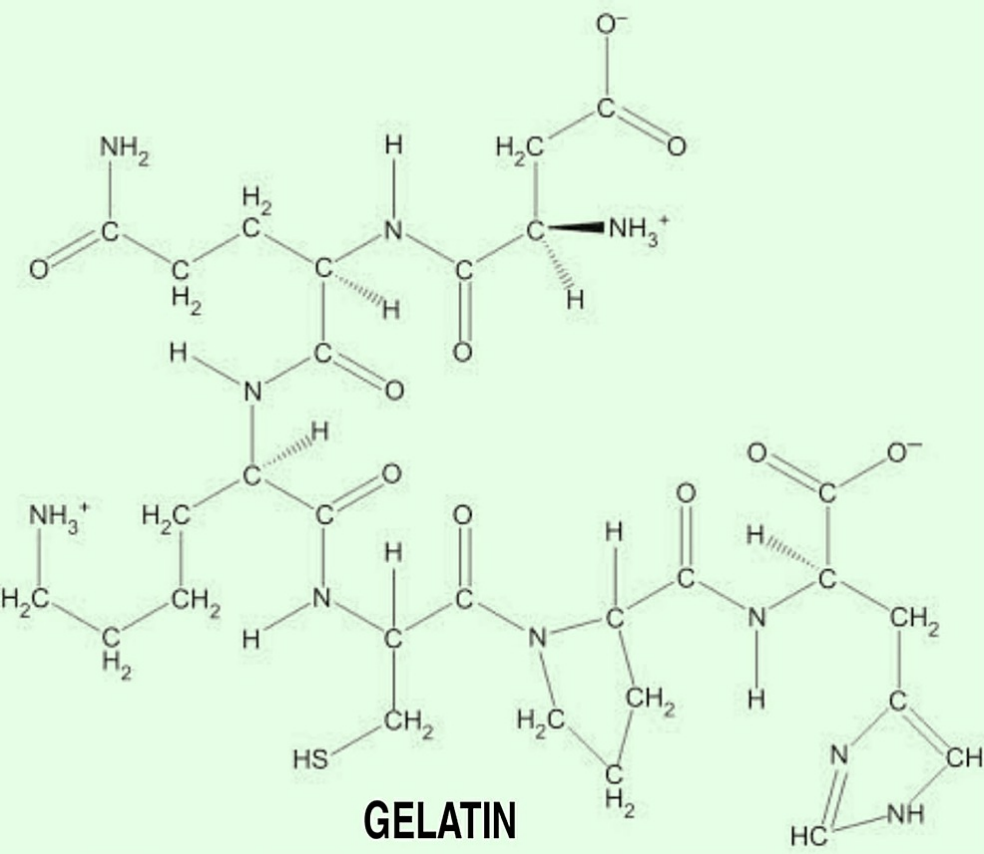
Chemical Structure of Gelatin

Gelatin is a linear molecule that is obtained by breaking collagen. Being a natural substance, it is not toxic, and is low in immunogenic properties, hydrophilic, and highly degradable, which makes it a special polymer. Before printing gelatin, it is combined with culture media to make it denser [21-26]. Many agents, such as hormone growth-promoting factors, can be crosslinked with gelatin molecules. Heparin, at the time of gelation, and other naturally occurring polymers such as hyaluronic acid, agarose, fibrin, collagen, and chitin will increase its mechanical strength and compatibility [27]. The combination of gelatin with synthetic polymers in the presence of UV light has led to the formation of GeIMA, which, when used in combination with HAMA (HAMA-GeIMA), will increase its mechanical strength and compatibility. It has been proven that the 1:4 ratio of GeIMA:HAMA is an adequate ratio to increase the polymer formed compatibility. Another combination of gelatin is crosslinking of gelatin with chemical agents such as calcium chloride to improve the stability and degradation properties of gelatin hydrogels such as gelatin-fibrin or gelatin-alginate combination [28,29]. The chemical structure of gelatin is given in Figure 6.

Fibrin

**Figure 7:**
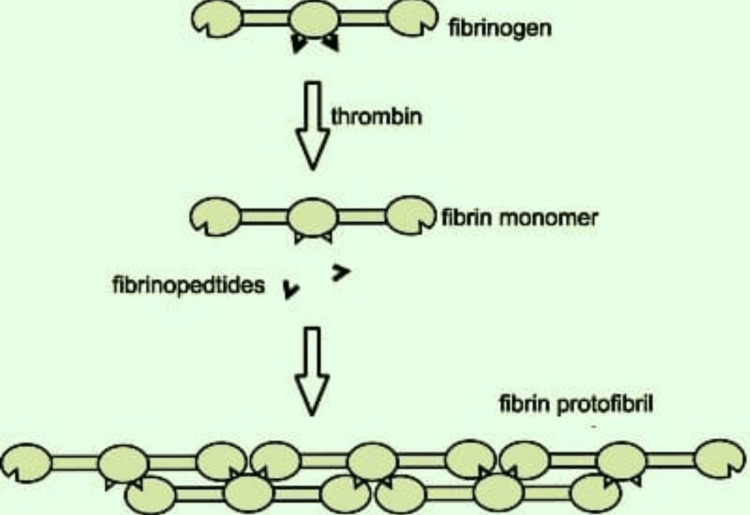
Diagram Illustrating Formation of Fibrin

Fibrin is a natural polymer as it is formed in blood in the presence of thrombin due to the rapid polymerization of fibrinogen [30]. Although fibrin has been found superior in its properties such as compatibility compared to other natural polymers to increase its efficacy, it is combined with other natural polymers to overcome its low strength, less viscosity, high degradation, and gelation properties when used alone [31]. The recent trend is to combine natural polymers and crosslink them using chemical agents to form a hybrid type of polymers in various combinations such as gelatin-chitosan-alginate-fibrinogen and gelatin-hyaluronic acid-glycerol-fibrin. This combination helps create a more stable structure that can print quickly, and those models can survive longer in the body's environment. Fibrin and its combination with other polymers are being used in bioprinting of skin, which will be helpful in early wound closure in many cases and early regeneration of tissue and its vasculature [32]. The formation of fibrin is illustrated in Figure 7.

Chitosan

**Figure 8:**
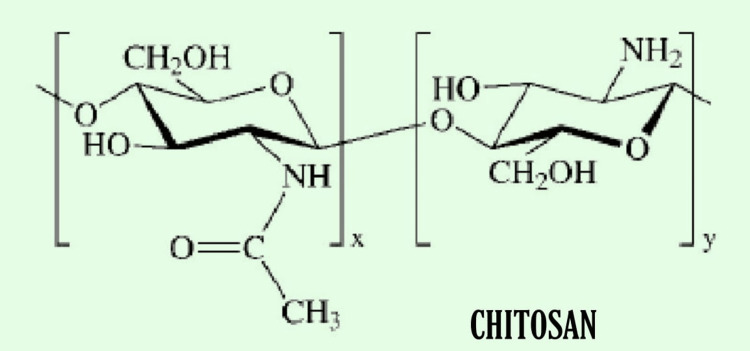
Chemical Structure of Chitosan

Chitosan is usually derived from shrimp shells and is formed from the hydrolysis of chitin. Like other natural polymers, it is low in strength and has degradable properties; therefore, a similar combination of crosslinking with chemical agents is done with collagen, alginate, and gelatin to increase its viscosity and biodegradability, and to make it more compatible, it is used to repair rigid structure such as skin, bone, and cartilage [33-38]. The chemical structure of chitosan is given in Figure 8.

Synthetic Polymers

Synthetic polymers are made artificially by humans in a laboratory using chemicals in the appropriate environment required for their production; they are high in strength and resistance. The main advantage of synthetic polymers is that we can modify them easily as they can withstand changes in temperature and pH and can be processed according to our needs due to their increased resistance and mechanical strength. Since the gelation temperature of synthetic polymers is shallow compared to natural polymers with a very high melting temperature, they are very suitable for models for 3D bioprinting; therefore, formed polymers are inert, are difficult to degrade, and have a high tensile strength.

Polyethylene Glycol

**Figure 9:**
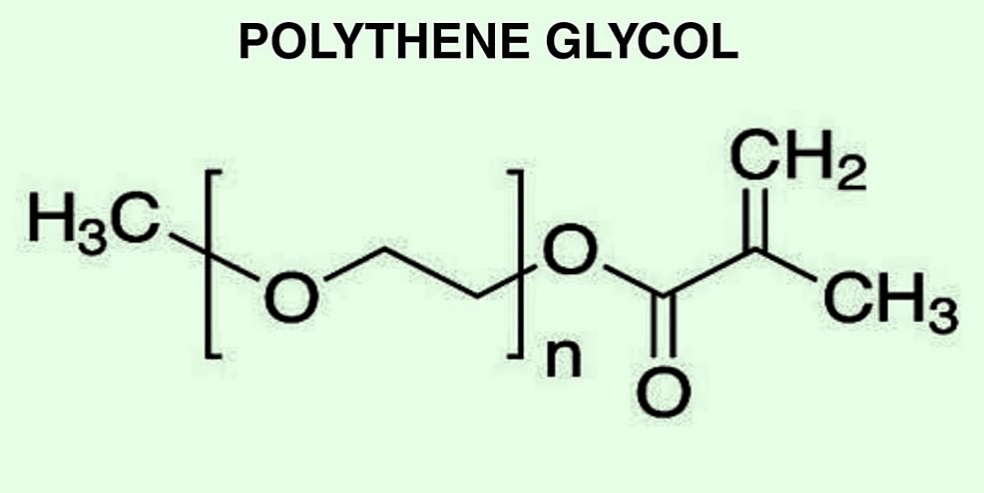
Chemical Structure of Polyethylene Glycol

Polyethylene glycol is a linear synthetic polymer that is compatible, is low in immunogenicity, and has a high affinity for water, making it well qualified for bioprinting. Another name for polyethylene glycol is polyethylene oxide [39,40]. Polyethylene glycol cannot adhere appropriately to the cells; it is crosslinked with other molecules such as carboxyl group, acrylate, or thiol group to make it more suitable for use in the repair of soft tissues. Polyethylene glycol can also be polymerized in the presence of UV light to increase cell encapsulation rate and its mechanical strength. Using the Inkjet bioprinting technique, PEG has also been crosslinked with GeIMA to increase its strength for the bioprinting of rigid structures such as cartilage and bone [41,42]. Since polyethylene glycol is not degraded on its own, hydrolytic blocks such as polycaprolactone and PGA is used to increase its degradation rate. The chemical structure of polyethylene glycol is given in Figure 9.

Polycaprolactone

**Figure 10:**
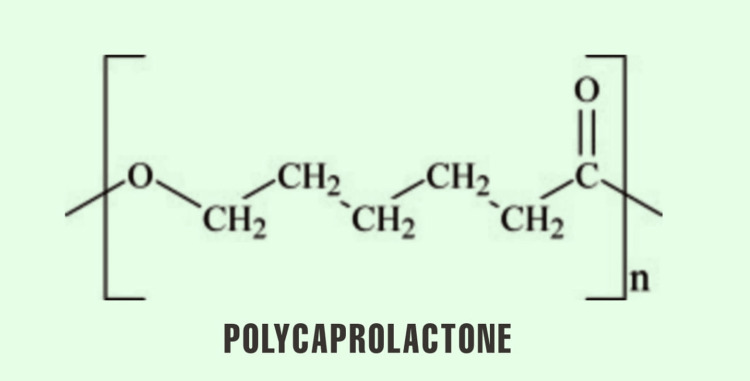
Chemical Structure of Polycaprolactone

Polycaprolactone is a partially crystalline polymer that can be easily degraded naturally in our body [43]. It is a thermoplastic polymer produced at the temperature of -60°C when combined with other agents to change its mechanical structure and degradation rate. It can be called an ideal material to be used in fused deposition modelling technology of 3D bioprinting [44,45]. As it is done in all other synthetic polymers, polycaprolactone is crosslinked with other bioagents such as polycaprolactone-alginate to increase its cell adhesive property for regeneration of cartilage. Polycaprolactone has also been combined with GeIMA using UV light to increase the strength and stability of the scaffold. GeIMA concentration is proportional to the hardness of the scaffold and is widely used in cartilage and bone regeneration [46]. Other uses of polycaprolactone are to form sutures and in devices such as drug delivery system [47]. The chemical structure of polycaprolactone is given in Figure 10.

Polyurethane

**Figure 11:**
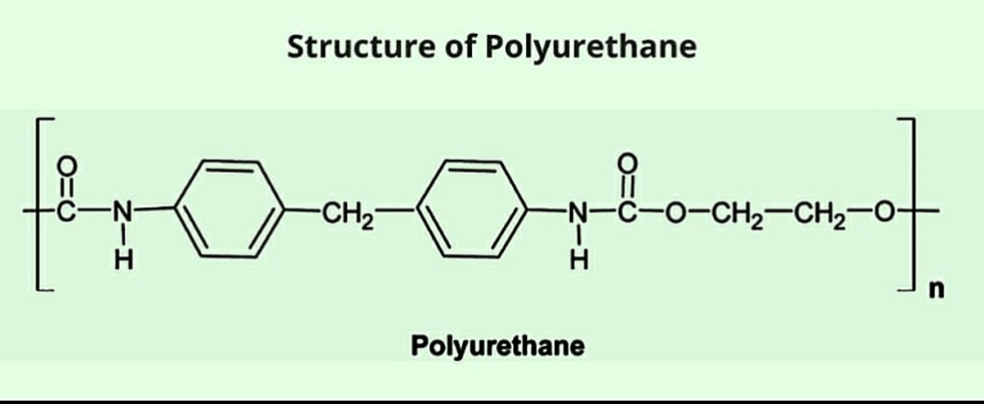
Chemical Structure of Polyurethane

Polyurethane is a linear biodegradable polymer that shows outstanding compatibility and mechanical properties [48]. Polyurethane, when used alone, is inert and cannot be degraded. Therefore, it is crosslinked with other materials to increase its compatibility and stability. Waterborne polyurethane is one such type that removes its problem of temperature and pH dependency, which is mainly dependent on its short segment (diol segment). Waterborne polyurethane is now used to repair chondrocytes and nerve cells [49-51]. Polyurethane has also been crosslinked with other bioagents such as adipose stem cell-fibrin-alginate-gelatin and cryoprotectant to protect against the damage from low temperatures to synthesize them. Another form of polyurethane is an elastic variety of polyurethane, which has been widely used for nerve repair and vascular repair conduits. Combining polyurethane with polycaprolactone and polyethylene glycol increases its mechanical strength, stability, compatibility, and biodegradability [52]. The chemical structure of polyurethane is given in Figure 11.

Polylactic-Co-Galactic Acid

**Figure 12:**
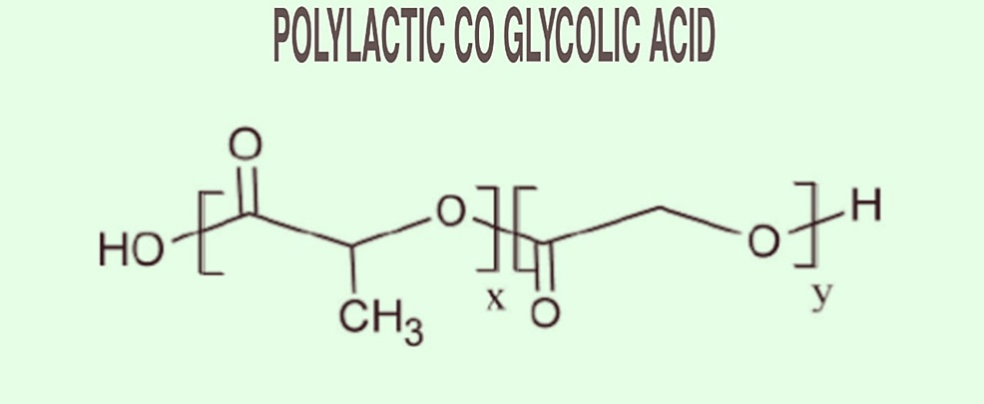
Chemical Structure of Polylactic-Co-Glycolic Acid

Polylactic-co-glycolic acid is formed using two polymers, lactic acid and glycolic acid, by copolymerization. It is usually seen that the transition temperature of polylactic-co-glycolic acid is around 40-60°C, and glycolic acid and lactic acid are used in the ratio of 1:3 [53]. It has been observed that the degradation rate of polylactic-co-galactic acid depends on the concentration of glycolic acid used while synthesizing. Polylactic-co-galactic acid is mainly used where high mechanical support is required [54]. It can also be combined with other agents such as growth-promoting factors or adipose stem cells to make it more useful and compatible for making the complicated structure of 3D bioprinted organs. PLGA can also be synthesized at low temperatures to create a complex organ structure with fibrin hydrogel to act as a native organ when transplanted [55]. The chemical structure of poly-co-galactic acid is given in Figure 12.

Recent advances in bioprinting technology

The application of the internet of things (IoT) with the technology of bioprinting has led to breakthroughs in surgical techniques [56]. The ultimate goal is to break the chain of years and years of waiting for a donor organ and to print an entire organ that will be structurally and functionally similar. The main organs on focus to print are our heart, bone, skin, cartilage, and tendon. Besides focusing on printing an entire organ, 3D bioprinting has been used in various other branches, which have been described in Table 3 [57-60].

**Table 3:** Summary of Application of Bioprinting in Different Industries

Industries	Uses
Dental	Crowns, filling, implants, fixtures
Pharmacy	Drug delivery
Medicine	Pharmacy, prosthetics, hearing aids, orthopedic screws/plates
Food	Cookie, candy, pizza
Automobile industry	Prototypes, spare parts

## Conclusions

Even though there is still a long road ahead of us to print an organ, this cutting-edge technology has shown a promising potential that will change the lives of thousands of people dying every day because of the need for a donor organ. However, implanting a printed organ in a human body is still scary for many people. If successful, it will solve many problems, such as a long waiting list for a transplant and issues of organ rejection, and will completely change the face of medicine. Since, at present, there are not enough biomaterials that can be used in 3D bioprinting, there is a high need for research in this matter as this shows the potential of saving the lives of many patients who require a transplant. Still, in its early phases, bioprinted organs have already proved functional in labs, but there is a long road in front of us until they will be transplanted into an actual human body.
